# Phacoemulsification after trabeculectomy in uveitis associated with Vogt-Koyanagi-Harada disease: intermediate-term visual outcome, IOP control and trabeculectomy survival

**DOI:** 10.1186/s12886-022-02438-3

**Published:** 2022-05-09

**Authors:** Faisal A. Almobarak, Ali H. Alharbi, Ibrahim Aljadaan, Hassan Aldhibi

**Affiliations:** 1 Department of Ophthalmology, College of medicine, King Saud University, P.O. Box 245, Riyadh, 11411 Saudi Arabia; 2 Glaucoma Research Chair, King Saud University, Riyadh, Saudi Arabia; 3 Glaucoma Division, King Khaled Eye Specialist Hospital, Riyadh, Saudi Arabia; 4 Uveitis Division, King Khaled Eye Specialist Hospital, Riyadh, Saudi Arabia; 5 Department of Ophthalmology, King Abdullah Medical City, Makkah, Saudi Arabia

**Keywords:** Glaucoma, Uveitis, Trabeculectomy, Cataract, Vogt-Koyanagi-Harada disease

## Abstract

**Purpose:**

To evaluate the visual outcome, intraocular pressure control and trabeculectomy survival after phacoemulsification in eyes with prior trabeculectomy in uveitis associated with Vogt-Koyanagi-Harada disease (VKH).

**Design:**

Retrospective comparative study.

**Methods:**

Eyes with uveitic glaucoma associated with VKH who underwent mitomycin C (MMC)-enhanced trabeculectomy were included. Eyes were divided into two groups: the first study group included eyes that later underwent cataract surgery in the form of phacoemulsification, and the second control group included eyes that did not have cataract surgery. The main outcome measures were changes in the visual acuity, intraocular pressure (IOP), the number of antiglaucoma medications, IOP control and trabeculectomy survival.

**Results:**

There were no significant differences in the final visual acuity (0.78 (±0.9) and 0.92 (±1.1), *p* = 0.80)) nor IOP (14.21 mmHg (±5.8) and 12.16 mmHg (±6.1), *p* = 0.29), but there was a difference in the antiglaucoma medications (1.58 (±1.5) and 0.53 (±1.0), *p* = 0.02) between the study and control group, respectively. There was no difference in the overall trabeculectomy survival (*p* = 0.381, Log Rank), but more eyes in the study group converted to qualified success after phacoemulsification and required more medications to control the IOP.

**Conclusion:**

Phacoemulsification after trabeculectomy seems to be a safe procedure in eyes with combined vision threatening complications of VKH, although the visual improvement was limited. Nevertheless, more medications were required to control the IOP, resulting in less absolute and more qualified trabeculectomy success. Therefore, patient counseling before surgery is essential.

## Background

Vogt-Koyanagi-Harada disease (VKH) is a T-cell mediated autoimmune disease against antigen or antigens associated with melanocytes which commonly affects pigmented races such as Asians, Native Americans, Hispanics, Indians and Middle easterners [1, 2]. The acute disease phase is characterized by bilateral granulomatous panuveitis and exudative retinal detachment, while the chronic phase includes recurrent granulomatous anterior uveitis with typical sunset glow fundus and chorioretinal atrophy [3, 4]. Glaucoma, subretinal neovascular membranes and fibrosis are vision-threatening complications recognized to occur mostly in the chronic recurrent phase and associated with poor visual outcome [5–8]. Cataract is the most common complication of VKH accounting for 25% of visual loss and has a prevalence of 40%. In addition, cataract and glaucoma surgeries are the most common surgeries carried out for patients with VKH [2, 7–9].

Several studies have reported favorable visual outcome of cataract surgery in VKH patients. Such an outcome can be achieved by controlling the intraocular inflammation in the absence of other vision-threatening complications of VKH [9–11]. However, such an outcome is unknown in patients who had combined glaucoma and cataract. Given that trabeculectomy is one of the commonly performed surgeries needed to control glaucoma in VKH patients and an important cataractogenic factor in uveitic glaucoma [7, 8, 12, 13], we were interested to evaluate the visual outcome, intraocular pressure (IOP) control and trabeculectomy survival after phacoemulsification in patients with previous trabeculectomy.

## Methods

### Patients

We reviewed medical records of patients with uveitic glaucoma associated with VKH who underwent MMC-enhanced trabeculectomy at King Khaled Eye Specialist Hospital, Riyadh, Saudi Arabia between 2005 and 2017. The diagnosis of VKH disease was based on the Revised International Diagnostic Criteria [14]. The inclusion criteria were (i) controlled IOP of < 21 mmHg with or without antiglaucoma medications, (ii) phakic eyes, (iii) no previous history of a pressure-lowering procedure other than a single MMC-enhanced trabeculectomy and (iv) a minimum follow-up time of 1 year. We identified 45 eyes of 23 patients out of which 19 eyes underwent phacoemulsification with intraocular lens implantation later on. Eyes were divided into 2 groups: the study group included the 19 eyes that underwent phacoemulsification after trabeculectomy and the control group included eyes that underwent only trabeculectomy surgery. We followed the same methods as described by Almobarak et al. [15] The control pairs were matched according to age, gender, trabeculectomy and phacoemulsification (study eyes)/admittance to the study (control eyes) time, best corrected visual acuity (BCVA), IOP and number of glaucoma medications. To match the study-control pairs with respect to trabeculectomy and phacoemulsification (study eyes)/admittance to the study time (control eyes), the time interval between trabeculectomy and phacoemulsification and between phacoemulsification and the final follow-up was calculated for the study group. For the control group, the study entry time was determined as the best matching time for that control eye with study eye considering the time between trabeculectomy and phacoemulsification of the study eye. Time interval between trabeculectomy and study entry, and then between study entry and final follow-up was determined. These times periods were then utilized to match the control and study eyes. Ultimately, 19 study eyes and 19 control eyes were included in the study, while the remaining eyes were excluded. The study was approved by the Ethics Review Board of King Khaled Eye Specialist Hospital, part of a retrospective study on uveitic glaucoma. All procedures adhered to the tenets of the declaration of Helsinki, and all patients gave written informed consent.

### Surgical technique

All patients were evaluated by uveitis specialist before surgery. Phacoemulsification was performed by experienced surgeons credentialed for the procedure. The temporal corneal incision was performed to avoid the filtering bleb superiorly. Any posterior synechiae were released with viscoelastic material or a cyclodialysis spatula before phacoemulsification. Iris retractors were employed when needed to dilate the pupil. A foldable intraocular lens (IOL) was implanted in the bag, and the wound was hydrated or closed with 10–0 nylon sutures. Finally, subconjunctival injections of antibiotics and steroids were administered. The patients were checked postoperatively depending on the stability of their condition. After the surgery, all of the patients were treated with topical ofloxacin eye drops four times daily for 2 weeks and hourly prednisolone acetate 1% eye drops; and the dosage was gradually adjusted on weekly basis and tapered to the preoperative maintenance dosage by the uveitis specialist. Systemic immunosuppressive agents such as mycophenolate mofetil 1 g twice daily, or azathioprine 50 mg twice daily (1–3 mg/kg/day) if mycophenolate mofetil is not tolerated were given for 3 months to achieve the optimal control of the inflammation, and then tapered afterwards. While oral prednisone 1 mg/kg/day (average of 40 to 50 mg daily) was given for 1 week and then gradually tapered on a weekly basis to the preoperative level. Postoperative monitoring was carried out by both the glaucoma and uveitis specialists.

### Data analysis

Data were collected whenever available and applicable for the following variables: age, gender, best corrected visual acuity (BCVA) converted into logarithm of minimal angle of resolution (logMAR) format, IOP, number of antiglaucoma medications and the need for subsequent pressure-lowering procedures to control the IOP after phacoemulsification. Good VA was defined as a BCVA of 20/40 or better, while poor VA was defined as a BCVA worse than 20/40. Grading of the anterior chamber cells was done following the standardization of uveitis nomenclature scheme [16]. Quiescence was defined as occasional cells or less, and recurrence of inflammation as a two-step increase to at least + 2 cells. The standardization of uveitis nomenclature for recurrence was followed and attributed to VKH if it occurred after 3 months from phacoemulsification [11]. All surgeries were performed after having a quiescence of at least 3 months. We evaluated variables between the 2 groups using the unpaired Student t-test, Mann-Whitney U test or Chi-square test, and within groups using the Wilcoxon pair test and paired Student t-test. We considered trabeculectomy as (i) complete success if the IOP was between 6 and 21 mmHg without antiglaucoma medications; (ii) qualified success if the IOP was between 6 and 21 mmHg with antiglaucoma medications; (iii) failure if the IOP was more than 21 mmHg on two visits despite maximum tolerated antiglaucoma medications or if an additional glaucoma procedure was needed to control the IOP. The cumulative probabilities of overall success, presented as percentage ± standard error (SE), were determined by Kaplan-Meier life table analysis for both groups and compared using the Mantel-Cox log-rank test. We presented variables as mean and standard deviation (SD), and considered *p* values of less than 0.05 to indicate statistical significance. Statistical analysis was carried out using SPSS version 23 (SPSS Inc., Chicago, IL).

## Results

Thirty-eight eyes of 22 patients, 10 males (45.5%) and 12 (54.5%) females, were included in this study. Ten eyes of 5 patients were included in each group. In the study group, 19 eyes underwent uneventful phacoemulsification with placement of a foldable IOL in the bag. Synechiolysis was performed in 13 eyes, two of them needed iris hooks as well to mechanically dilate the pupil, while the remaining 6 eyes did not need synechiolysis. The type of cataracts was posterior subcapsular cataract in 15 eyes (78.9%) and combination of nuclear sclerosis and posterior subcapsular cataract in 4 eyes (21.1%). There we no intraoperative complications. For the study eyes, the mean time between trabeculectomy and phacoemulsification was 33.21 months (±33.3), and the mean follow-up time was 45.75 months (±36.4). In the control group, the mean time between trabeculectomy and study entry was 35.79 months (±36.6), and the mean follow-up time was 50.33 months (±38.9); these differences between the 2 groups were not statistically significant (*p* = 0.82 and 0.71, respectively, Mann-Whitney U test). The mean ages of the patients in the study and control groups were 29.21 (±9.1) and 33.61 years (±11.0), respectively (*p* = 0.19, Mann-Whitney U test) (Table 1). There were no significant differences in the mean preoperative LogMAR BCVA, IOP nor number of antiglaucoma medications between the study and control groups (*p* = 0.90, 0.14 and 0.27, respectively). On the last follow-up, there were no differences in the mean LogMAR (0.78 (±0.9) and 0.92 (±1.1), *p* = 0.8) nor IOP (14.21 mmHg (±5.8) and 12.16 mmHg (±6.1), *p* = 0.29), but there was a difference in the antiglaucoma medications (1.58 medications (±1.5) and 0.53 medications (±1.0), *p* = 0.02) in the study and control groups, respectively. During the whole follow-up period, more eyes in the study group had good VA (20/40 or better) after phacoemulsification than the control group, although not statistically significant. On the last visit, the VA improved by 2 lines or more in 10 eyes (52.6%) in the study group, one eye (5.3%) improved by one line, one eye had the same VA (5.3%) and 7 eyes (36.8%) had worse VA. Factors contributing to such deterioration in VA include pre-existing advance glaucomatous damage, macular edema and subretinal neovascular membranes and fibrosis. When the 2 groups were compared, there were no differences in IOP nor logMAR throughout the follow-up period. Nevertheless, there was a difference in the mean number of antiglaucoma medications on all postoperative visits except at 3 and 12 months. The intragroup analysis demonstrated significant differences in the mean number of antiglaucoma medications in the study group but none in the control group (Table 2). Two eyes in the control group developed recurrence of anterior chamber inflammation but none in the study group. Posterior capsular opacification developed in 6 eyes (31.6%) while fibrinous reaction occurred in one eye (5.3%) in the first week postoperatively and resolved within 2 weeks.

**Table 1:** Demographics and ocular history^a^

Variable	Study Group (*N* = 19)	Control Group (*N* = 19)	*P*-value
Age, years	29.21 (±9.1)	33.61 (±11.0)	0.19
Gender^b^			
Male (10)	2 (20.0%)	8 (80.0%)	0.09
Female (12)	7 (58.3%)	5 (41.7%)	
Pre-Trabeculectomy:			
LogMAR	0.68 (±0.5)	0.86 (±0.9)	0.10
Mean IOP before Trabeculectomy, mmHg	40.05 (±9.1)	39.16 (±8.4)	0.76
Mean number of antiglaucoma medications before Trabeculectomy	3.95 (±0.6)	3.74 (±0.7)	0.35
Concentration of MMC			
0.02% (0.2 mg/ml) MMC	13	9	0.16
0.04% (0.4 mg/ml) MMC	6	10	
Duration of MMC exposure (min)			
0.02% (0.2 mg/ml) MMC	3.23 (±0.9)	3.00 (±0.7)	0.54
0.04% (0.4 mg/ml) MMC	3.33 (±0.5)	3.10 (±0.3)	0.28
Time to cataract surgery/study entry, months	33.21 (±33.3)	35.79 (±36.6)	0.82
Gonioscopy status			
Open	14 (73.7%)	9 (47.4%)	0.09
Closed	5 (26.3%)	10 (52.6%)	
Follow-up, months	45.75 (±36.4)	50.33 (±38.9)	0.71

^a^Data are presented as mean (±SD) and frequencies (%). Numbers are per eyes

^b^Numbers are per patients

*P*-values were calculated using Student t-test, Mann-Whitney U test and Chi-Square test

**Table 2:** IOP, antiglaucoma medications, LogMAR and changes in BCVA^a^

Variable	Study Group (*N* = 19)	Control group (*N* = 19)	*P*-value
Baseline			
IOP, mmHg	14.05 (±4.3)	12.16 (±3.4)	0.14
No. of medications	0.42 (±0.7)	0.46 (±0.5)	0.27
LogMAR	0.74 (±0.6)	0.71 (±0.6)	0.90
BCVA 20/40 or better	4 (21.1%)	4 (21.1%)	0.90
BCVA worse than 20/40	15 (78.9%)	15 (78.9%)	
No. of eyes	19	19	
3 Months postoperative			
IOP, mmHg	14.73 (±4.3)	12.46 (±8.9)	0.92
(*P*-value)^b^	(*p* = 0.18)	(*p* = 0.31)	
No. of medications	0.53 (±0.6)	0.63 (±1.0)	0.76
(*P*-value)^b^	(*p* = 0.30)	(*p* = 0.11)	
LogMAR	0.68 (±0.2)	0.84 (±1.0)	0.55
(*P*-value)^b^	(*p* = 0.29)	(*p* = 0.70)	
BCVA 20/40 or better	9 (47.4%)	5 (26.3%)	0.16
BCVA worse than 20/40	10 (52.6%)	14 (73.7%)	
No. of eyes	17	19	
12 Months postoperative			
IOP, mmHg	17.00 (±6.8)	13.00 (±6.1)	0.25
(*P*-value)^b^	(*p* = 0.36)	(*p* = 0.27)	
No. of medications	1.08 (±1.6)	0.62 (±0.9)	0.16
(*P*-value)^b^	(*p* = 0.02)	(*p* = 0.21)	
LogMAR	0.78 (±0.6)	0.85 (±0.8)	0.81
(*P*-value)^b^	(*p* = 0.42)	(*p* = 0.32)	
BCVA 20/40 or better	10 (52.6%)	5 (26.3%)	0.29
BCVA worse than 20/40	9 (47.4%)	14 (73.7%)	
No. of eyes	13	15	
24 Months postoperative			
IOP, mmHg	13.71 (±3.6)	12.00 (±5.8)	0.42
(*P*-value)^b^	(*p* = 0.35)	(*p* = 0.91)	
No. of medications	1.00 (±1.2)	0.52 (±0.7)	0.04
(*P*-value)^b^	(*p* = 0.04)	(*p* = 0.54)	
LogMAR	0.72 (±0.8)	0.77 (±0.9)	0.69
(*P*-value)^b^	(*p* = 0.39)	(*p* = 0.40)	
BCVA 20/40 or better	10 (52.6%)	6 (31.6%)	0.21
BCVA worse than 20/40	9 (47.4%)	13 (68.4%)	
No. of eyes	9	11	
36 Months postoperative			
IOP, mmHg	14.80 (±1.3)	13.50 (±7.5)	0.71
(*P*-value)^b^	(*p* = 0.88)	(*p* = 0.75)	
No. of medications	1.00 (±1.7)	0.50 (±0.8)	0.02
(*P*-value)^b^	(*p* = 0.03)	(*p* = 0.74)	
LogMAR	0.79 (±0.7)	0.81 (±0.8)	0.51
(*P*-value)^b^	(*p* = 0.47)	(*p* = 0.29)	
BCVA 20/40 or better	9 (47.4%)	6 (31.6%)	0.30
BCVA worse than 20/40	10 (52.6%)	13 (68.4%)	
No. of eyes	6	6	
Last visit			
IOP, mmHg	14.21 (±5.8)	12.61 (±6.1)	0.29
(*P*-value)^b^	(*p* = 0.92)	(*p* = 0.91)	
No. of medications	1.58 (±1.5)	0.53 (±1.0)	0.02
(*P*-value)^b^	(*p* = 0.02)	(*p* = 0.17)	
LogMAR	0.78 (±0.9)	0.92 (±1.0)	0.69
(*P*-value)^b^	(*p* = 0.84)	(*p* = 0.59)	
BCVA 20/40 or better	9 (47.4%)	7 (36.8%)	0.37
BCVA worse than 20/40	10 (52.6%)	12 (63.2%)	

^a^Data are presented as mean (±SD)

^b^Compared with baseline value in the group

*P*-values are calculated using Student t-test, Mann-Whitney U test, Wilcoxon test and Chi-square test

**Fig. 1:**
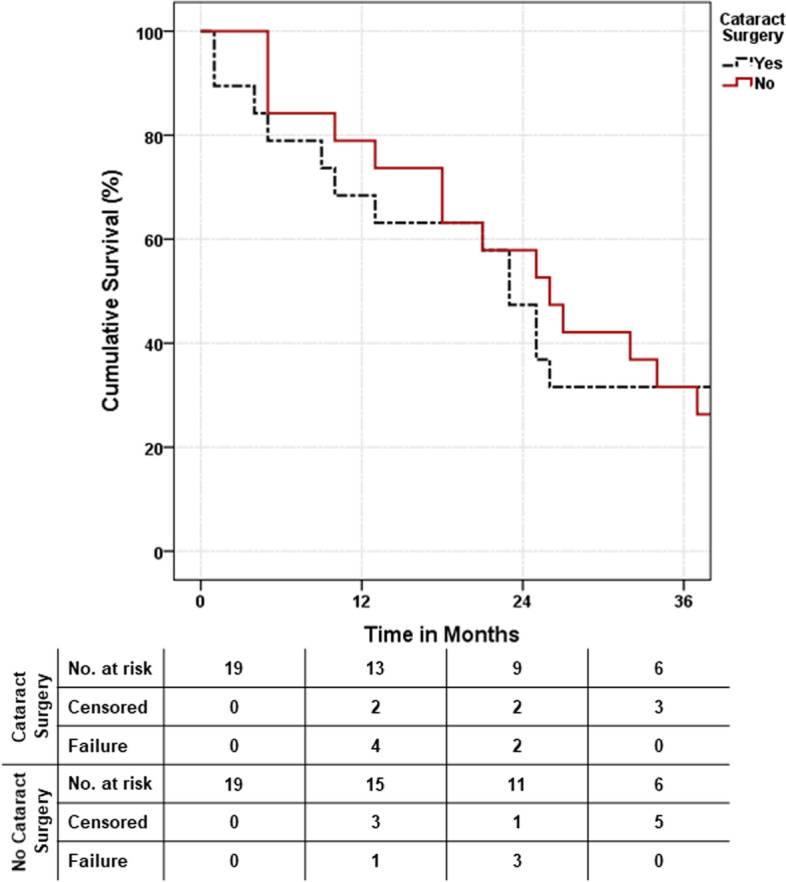
Kaplan-Meier survival curves showing the cumulative probability of success

The cumulative probabilities of overall success of the trabeculectomy for the study group were 68.4% (±10.7%), 47.4% (±11.5%) and 31.6% (±10.7%) compared with 78.9% (±9.4%), 57.9% (±11.3%) and 31.6% (±10.7%) in the control group at 12, 24 and 36 months after surgery, respectively; there was no significant difference in the trabeculectomy survival rate between the 2 groups (*p* = 0.381, Log Rank) (Fig. 1). The absolute success rate decreased from 78.9 and 84.2% to 31.6 and 52.6% on the last follow-up in study and control groups, respectively; whereas the qualified success rate increased from 21.1 to 36.8% in the study group but did not change in control group (15.8%). At the final follow-up, 7 eyes (36.8%) in the study group and 9 control eyes (47.4%) maintained their success status (either absolute or qualified success) (Table 3). The trabeculectomy failed in 6 eyes (31.6%) in each group:3 eyes in the study group needed additional glaucoma procedures to control the IOP: 2 eyes needed additional MMC-enhanced trabeculectomy and one eye needed tube surgery, compared with 4 eyes in the control group: 3 eyes needed MMC-enhanced trabeculectomy and one eye needed cyclophotocoagulation (Table 4).

**Table 3:** Success status and antiglaucoma medications in study and control groups^a^

	Baseline			Last follow-up		
	Study Group	Control Group	*P*-value**	Study Group	Control Group	*P*-value**
Success status			0.50			0.28
Absolute success	15 (78.9%)	16 (84.2%)		6 (31.6%)	10 (52.6%)	
(maintain status)	(5/15)	(8/16)				
(failed)	(5/15)	(6/16)				
Qualified success	4 (21.1%)	3 (15.8%)		7 (36.8%)	3 (15.8%)	
(maintain status)	(2/4)	(1/3)				
(failed)	(1/4)	Zero				
Failure	–	–		6 (31.6%)	6 (31.6%)	
Medications						
One or None	17 (89.5%)	19 (100%)	0.24	10 (52.6%)	15 (78.9%)	0.09
Two or more	2 (10.5%)	Zero		9 (47.4%)	4 (21.1%)	

^a^Data are presented as frequencies (%). Numbers are per eyes

**P-value calculated using Chi-Square test

**Table 4:** Eyes needed repeat glaucoma surgery

	Study Group (*N* = 19)	Control Group (*N* = 19)
First repeat surgery	3/19 (15.8%)	4/19 (21.1%)
Trabeculectomy + MMC	2	3
Tube surgery	1	0
Cyclophotocoagulation	0	1
Second repeat surgery	3/19 (15.8%)	1/19 (5.3%)
Trabeculectomy + MMC	0	Zero
Tube surgery	3	1
Cyclophotocoagulation	0	0
Third repeat surgery	0/19	1/19 (5.3%)
Trabeculectomy + MMC	0	0
Tube surgery	0	0
Cyclophotocoagulation	0	1

## Discussion

VKH is one of the most common uveitis entities in Saudi Arabia affecting mostly young adults. Glaucoma and cataract are among the most common complications in such entity [17, 18]. Several studies have reported a high frequency (23.1–51.6%) of cataract development and progression in uveitic glaucoma eyes after trabeculectomy which eventually required cataract surgery. Cataract surgery in trabeculectomized eyes with uveitis is challenging as a result of the high risk of postoperative inflammation and loss of IOP control. Nevertheless, decreased perioperative and long-term intraocular inflammation and improved microsurgical techniques have all resulted in better outcomes [19].

We have previously reported favorable outcome of phacoemulsification after trabeculectomy in uveitis [15]. However, the outcome in a major uveitis entity such as VKH is not well known. Patients included in the current study are of young age group, due to the existence of chronic inflammation, prolonged use of steroids and the disease prevalence in younger age group [18, 20, 21]. The VA improved in half the study group (almost 58%), mostly by 2 lines. Our results are less than those reported by Quek et al. [11] (82%), and Ganesh et al. [10] (80%). However, our group included patients with combined vision threatening complications of VKH, mainly glaucomatous disc damage and retinal pathologies associated with VKH including subretinal neovascular membranes and fibrosis and macular edema, accounting for poor vision in almost one third of eyes after phacoemulsification [11, 20]. The indication of cataract surgery was not only visually significant cataract, but also to allow better visualization of the posterior pole to monitor any uveitis activity, especially in patients with cataract and synechia. In a meta-analysis done by Mehta et al. [22], patients with posterior uveitis such as VKH were found to have poorer visual results than those with other uveitis entities due to chorioretinal injury. However, such eyes may have visual improvement, even if not to 20/40 or better. Moreover, In the presence of cataract in uveitis with vision threatening complications, the goal of surgical intervention is not only visual rehabilitation, but also to have a better visualization of the retina and optic nerve and monitor progression.

Phacoemulsification less than 1 year after trabeculectomy can affect trabeculectomy function [23, 24]. In the current study, the mean time to phacoemulsification was around 3 years, which favors bleb survival. All eyes were at quiescent stage before phacoemulsification by 3 months, and only 2 eyes in the control group developed recurrence of anterior chamber inflammation but none in the study group. Three months of quiescence stage, the better understanding and control of inflammation and the close follow-up are important contributing factors for the prevention of recurrence of inflammation. Although there was no significant difference in the trabeculectomy survival rate between both groups, eyes that underwent phacoemulsification required more medications to control the IOP than the control eyes, and therefore more eyes converted from absolute to qualified success which is consistent with our previous report [15]. Phacoemulsification increases blood-aqueous barrier permeability and induces an inflammatory response. The filtration of inflammatory mediators through trabeculectomy flap results in macrophage and fibroblast activation, aggregation, collagen synthesis and deposition and eventual scarring. Furthermore, an increased uveoscleral outflow results in less aqueous drainage through filtering bleb and a high IOP [15]. The presence of such low-grade inflammation and enriching the bleb area by inflammatory cells can affect trabeculectomy function, increase the IOP and therefore, require more medications. Vigorous use of topical steroids is needed to control such an inflammatory process. However, steroids induced glaucoma could contribute as well for IOP elevation in uveitic glaucoma, depending on the patient susceptibility, dose, duration, type of medication and route of administration [25, 26]. The use of 5-flurouracil (5-FU) has been described before, and suggested to have a protective role in bleb function by inhibiting wound healing and scarring potential [27]. It would be interesting to explore the potential role of 5-FU while decreasing the burden of steroids.

Differences in the trabeculectomy success rates between the study and control groups were observed during the first 24 months after phacoemulsification. In their study of eyes after cataract surgery, Ehrnrooth et al. reported lower success rates and higher failure after a mean follow-up of 25.3 months compared with our study group [19]. Inal et al. reported no difference in the percentage of eyes considered an absolute success, qualified success and failure between eyes that underwent phacoemulsification and control eyes after 26 months follow-up [28]. Nishizawa et al. reported poor prognosis of IOP control after post-trabeculectomy phacoemulsification in uveitic glaucoma [24]. The current study revealed less IOP control after phacoemulsification in VKH, requiring antiglaucoma medications to control the pressure and therefore, changing the status from absolute to qualified success or increasing the medications burden which is in line with our previous study. Such differences in IOP control can be attributed to the underlying glaucoma and uveitis entities, and the difference in follow-up period, which can be influenced by the natural course of trabeculectomy. Both groups had progressive reduction in the cumulative probability of overall success for both groups, which is again most likely due to the natural course of trabeculectomy, the presence of low-grade inflammation related to uveitis, and is consistent with our previous report [15]. It is worth mentioning that more eyes in the study group had less MMC concentration during trabeculectomy. However, previous studies did not find significant difference in trabeculectomy function among different MMC concentrations [29, 30].

## Conclusions

This study has some limitations which include retrospective design, small sample sizes and offers an intermediate-term follow-up result. Collected data were limited to what were available in medical records. However, it represents the first study to evaluate the outcome of phacoemulsification after trabeculectomy in VKH patients. In conclusion, phacoemulsification resulted in higher number of medications to maintain a controlled IOP in glaucomatous VKH eyes. Such change in IOP control did not affect the overall survival. Visual outcome after phacoemulsification is governed by the severity of pre-existing glaucoma and retina related complications. Therefore, counselling the patient about such potential outcomes is advisable.

## Data Availability

All data generated or analyzed during this study are included in this published article.

## Abbreviations

IOP: Intra-ocular pressure
VKH: Vogt-Koyanagi-Harada Disease
MMC: Mitomycin C
